# Supplementary material for: Comparative Genomic Analysis Reveals Potential Pathogenicity and Slow-Growth Characteristics of Genus Brevundimonas and Description of Brevundimonas pishanensis sp. nov
Source: Microbiol Spectr. 2022 Apr 13;10(2):e02468-21. doi: 10.1128/spectrum.02468-21 (PMC9045160; doi:10.1128/spectrum.02468-21)
Supplement: SUPPLEMENTAL FILE 1 — Fig. S1-S4; Tables S1-S7. Download spectrum.02468-21-s001.pdf, PDF file, 1.5 MB [file spectrum.02468-21-s001.pdf]

Supplementary File

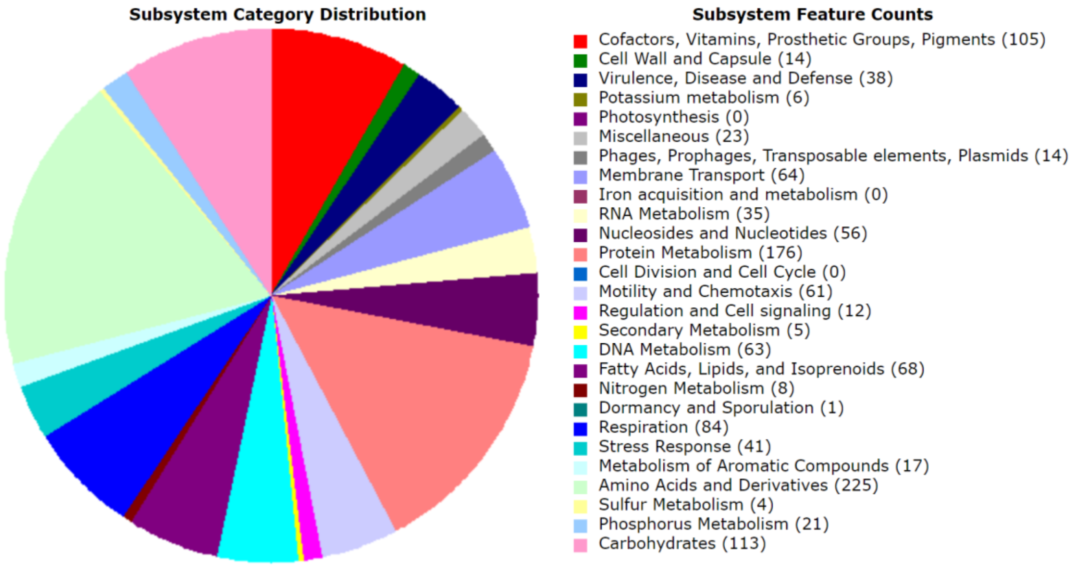

Fig. S1 Genomic annotation features in subsystems of *Brevundimonas* spp.

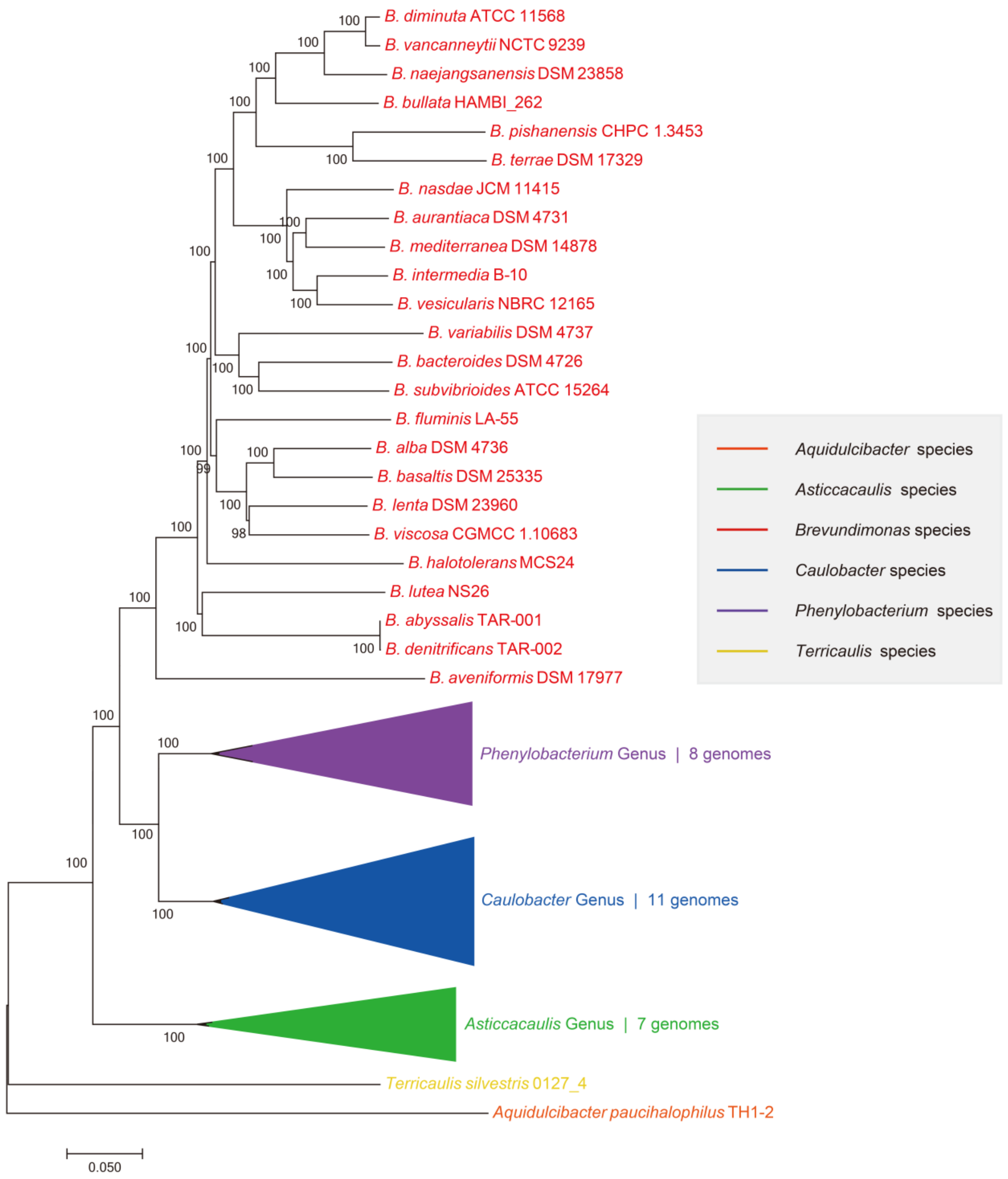

Fig. S2 Neighbor-joining tree based on genomic single-copy homologous gene sequences of the genus *Brevundimonas* in the entire *Caulobacteraceae* family. Colored lines represent different species. Bootstrap values over 70%, based on 1000 bootstrap resamplings, were shown at branch nodes. The horizonal bar represented 0.05 substitution per nucleotide site.

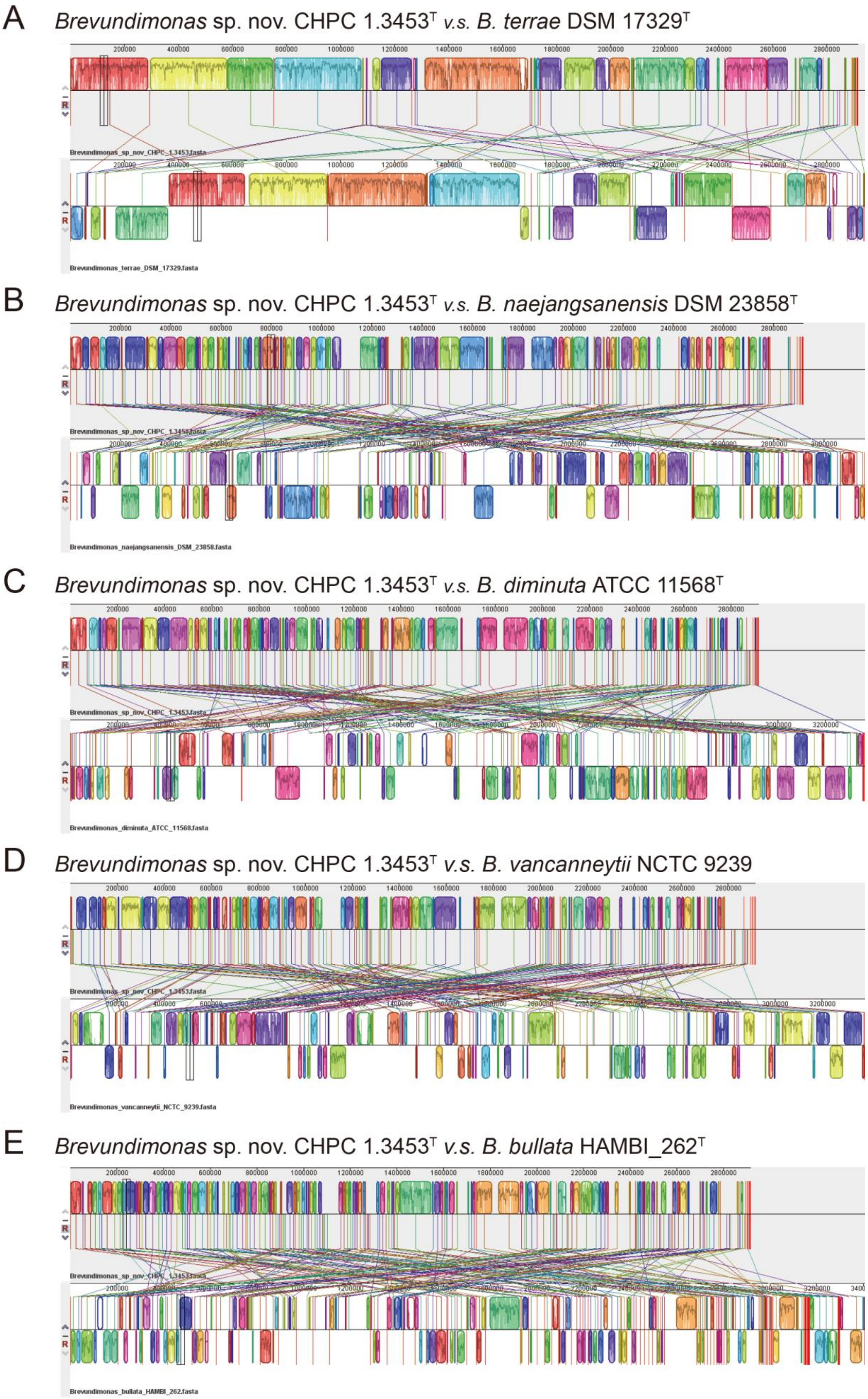

Fig. S3 Genomic structures comparison of the novel strain CHPC 1.3453^{T} with other *Brevundimonas* spp.

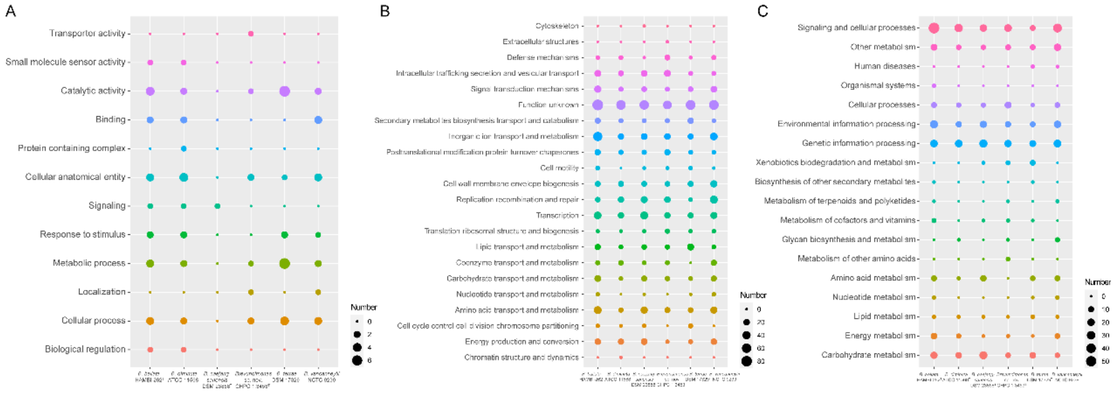

Fig. S4 Gene function annotation of unique genes among genomes in the *Clade V*. (A) GO annotation of unique genes found in six *Brevundimonas* genomes. (B) COG annotation of unique genes found in six *Brevundimonas* genomes. (C) KEGG annotation of unique genes found in six *Brevundimonas* genomes.

Table S1: Genomic overview of publicly available *Brevundimonas* strains analyzed in this study

No.	Species	Strain	BioSample	Size(Mb)	GC %	WGS	Scaffolds	CDS	Genes	rRNA	tRNA	other RNA	Isolation source	Geographic location	Collection year/Published year
1	*Brevundimonas abyssalis*	TAR-001^T	SAMD00008582	2.98	68.2	BATC01	128	2528	3062	3	41	4	Sediment	Japan	- / 2013
2	*Brevundimonas alba*	DSM 4736^T	SAMN13173387	3.06	68.6	JAATJM01	2	2976	3049	3	44	3	Soil	United Kingdom	- / 1999
3	*Brevundimonas aurantiaca*	DSM 4731^T	SAMN13173390	3.29	67.5	JACHOQ01	22	3149	3224	3	46	4	-	-	- / 1999
4	*Brevundimonas aveniformis*	DSM 17977^T	SAMN02440608	2.58	65.1	AUAO01	6	2559	2628	3	43	4	Sludge; Wastewater	South Korea	- / 2007
5	*Brevundimonas bacteroides*	DSM 4726^T	SAMN02841166	3.22	68.2	JNIX01	15	3148	3225	3	44	3	Aquatic; Fresh water; Lake	USA	1959 / 1999
6	*Brevundimonas basaltis*	DSM 25335^T	SAMN14908468	2.65	68.5	JACHFZ01	8	2631	2703	3	43	4	Black sand	South Korea	- / 2010
7	*Brevundimonas bullata*	HAMBI _262^T	SAMN09428652	3.41	67.0	QLLC01	85	3175	3285	3	48	4	-	-	1980 / 2009
8	*Brevundimonas denitrificans*	TAR-002^T	SAMD00094821	3.23	68.2	BEWU01	44	3147	3349	3	48	4	Sediment	Japan	2006/2014
9	*Brevundimonas diminuta*	ATCC 11568^T	SAMN02469913	3.37	66.3	ADUI01	8	3151	3398	3	47	4	Fresh water	-	- / 1994
10	*Brevundimonas fluminis*	LA-55^T	SAMN10390089	2.64	70.3	RQWJ01	10	2692	2761	3	43	4	River water	South Korea	2017 / 2020

11	*Brevundimonas halotolerans*	MCS24^{T}	SAMN09770276	2.54	66.9	QTTA01	24	2521	2600	3	44	3	Marine	USA	1987 /2010
12	*Brevundimonas intermedia*	B-10	SAMN10424492	3.34	66.5	SPVH01	7	3223	3311	6	49	4	Pond water	-	- / 1999
13	*Brevundimonas lenta*	DSM 23960^{T}	SAMN13173499	3.36	68.8	JACIDM01	6	3222	3292	3	43	4	Soil	South Korea	- / 2007
14	*Brevundimonas lutea*	NS26^{T}	SAMN09768131	2.98	68.4	QUOQ01	9	2858	2950	3	44	4	Sediment	China	2014 / 2019
15	*Brevundimonas mediterranea*	DSM 14878^{T}	SAMN13172971	3.41	67.0	JACIDA01	7	3231	3319	3	49	4	Seawater	France	1995 / 2005
16	*Brevundimonas naejangsanensis*	DSM 23858^{T}	SAMN02441231	3.16	66.6	ATXN01	10	2982	3080	9	53	4	Soil	South Korea	- /2009
17	*Brevundimonas nasdae*	TPW30	SAMN03257121	3.41	66.0	JWSY01	85	3289	3418	3	47	4	Russian space laboratory Mir	Japan	1997 / 2004
18	*Brevundimonas subvibrioides*	ATCC 15264^{T}	SAMN00007485	3.45	68.4	-	1	3306	3379	6	46	3	Fresh water	-	- / 1999
19	*Brevundimonas terrae*	DSM 17329^{T}	SAMN12025163	2.94	60.8	JAASQT01	13	2760	2835	3	49	4	Soil	South Korea	- / 2006
20	*Brevundimonas vancanneytii*	NCTC 9239	SAMEA3121333	3.38	67.3	-	1	3065	3353	6	51	4	Patient, blood	Germany	- /2010
21	*Brevundimonas variabilis*	DSM 4737^{T}	SAMN13172606	3.40	65.3	JACHOR01	8	3224	3302	6	46	3	Pond water	-	- / 1999
22	*Brevundimonas vesicularis*	NBRC 12165^{T}	SAMD00047217	3.36	66.3	BCWM01	42	3248	3334	3	44	4	Urinary-bladder epithelium of leech,	-	1953/1994

													Hirudo medicinalis		
23	*Brevundimonas viscosa*	CGMCC 1.10683^T	SAMN05192570	3.00	70.4	FOZV01	19	3060	3151	3	43	4	Soil	China	- / 2012
24	*Brevundimonas pishanensis*	CHPC 1.3453^T	SAMN23180853	2.92	61.6	JAJKBG01	17	2788	2875	3	47	4	Stool	China	2018/-

Note: T represents the tpye strain of corresponding *Brevundimonas* species

Table S2: Reference sequences of 16S rRNA gene among type strains analyzed in this study

No.	Species	Strain	Accession No.
1	*Asticcacaulis excentricus*	DSM 4724^T	AJ247194
2	*Brevundimonas abyssalis*	TAR-001^T	BATC01000012
3	*Brevundimonas alba*	DSM 4724^T	AJ227785
4	*Brevundimonas albigilva*	KACC 18249^T	KC733808
5	*Brevundimonas aurantiaca*	DSM 4731^T	AJ227787
6	*Brevundimonas aveniformis*	KCTC 12609^T	AUAO01000001
7	*Brevundimonas bacteroides*	DSM 4726^T	JNIX01000007
8	*Brevundimonas balnearis*	DSM 29841^T	LN651199
9	*Brevundimonas basaltis*	J22^T	EU143355
10	*Brevundimonas bullata*	IAM 13153^T	D12785
11	*Brevundimonas canariensis*	GTAE24^T	KX898252
12	*Brevundimonas denitrificans*	TAR-002^T	AB899817
13	*Brevundimonas diminuta*	ATCC 11568^T	GL883089

14	*Brevundimonas faecalis*	CS20.3^{T}	FR775448
15	*Brevundimonas fluminis*	LA-55^{T}	MH094643
16	*Brevundimonas halotolerans*	MCS24^{T}	QTTA01000016
17	*Brevundimonas humi*	CA 15^{T}	KY117472
18	*Brevundimonas intermedia*	ATCC 15262^{T}	AJ227786
19	*Brevundimonas kwangchunensis*	KCTC 12380^{T}	AY971368
20	*Brevundimonas lenta*	DS-18^{T}	EF363713
21	*Brevundimonas lutea*	NS26^{T}	KX601076
22	*Brevundimonas mediterranea*	V4.BO.10^{T}	AJ227801
23	*Brevundimonas mongoliensis*	R-10-10^{T}	MF436701
24	*Brevundimonas naejangsanensis*	DSM 23858^{T}	ATXN01000003
25	*Brevundimonas nasdae*	GTC 1043^{T}	AB071954
26	*Brevundimonas olei*	MJ15^{T}	GQ250440
27	*Brevundimonas poindexterae*	FWC40^{T}	AJ227797
28	*Brevundimonas staleyi*	FWC43^{T}	AJ227798
29	*Brevundimonas subvibrioides*	ATCC 15264^{T}	ADBM01000034
30	*Brevundimonas terrae*	KSL-145^{T}	DQ335215
31	*Brevundimonas vancanneytii*	LMG 2337^{T}	AJ227779
32	*Brevundimonas variabilis*	ATCC 15255^{T}	AJ227783
33	*Brevundimonas vesicularis*	NBRC 12165^{T}	BCWM01000033
34	*Brevundimonas viscosa*	CGMCC 1.10683^{T}	FOZV01
35	*Caulobacter fusiformis*	ATCC 15257^{T}	AJ227759
36	*Sphingomonas adhaesiva*	DSM 7418^{T}	KY927401

Table S3: Genomic overview of publicly available strains of the *Caulobacteraceae* family except for *Brevundimonas* genus

**Species**	**Strain**	**WGS accession no.**	**Assembly**	**Size (Mb)**	**G+C (mol%)**
Aquidulcibacter paucihalophilus	TH1-2	NCSQ01	GCF_002105465	3.74	55.7
Asticcacaulis biprosthecium	C19	ADUH01	GCA_000204015	5.41	60.7
Asticcacaulis excentricus	CB 48	-	GCA_000175215	4.3	59.5
Asticcacaulis benevestitus	DSM 16100	AQWM01	GCA_000376105	4.99	58.4
Asticcacaulis taihuensis	CGMCC 1.3431	FMTS01	GCA_900100255	3.97	59.8
Asticcacaulis tiandongensis	3.1105	SSBC01	GCA_006475605	3.22	56.5
Asticcacaulis solisilvae	BE327	JAGGMI01	GCA_017875235	5.01	61.7
Asticcacaulis endophyticus	KCTC 32296	BMZB01	GCA_014652575	3.95	56.8
Caulobacter vibrioides	NA1000	-	GCA_000022005	4.04	67.2
Caulobacter segnis	TK0059	-	GCA_003015125	4.66	67.7
Caulobacter rhizosphaerae	KCTC 52515	-	GCA_010977555	5.96	68.6
Caulobacter flavus	RHGG3	-	GCA_003722335	5.66	69.3
Caulobacter mirabilis	FWC 38	-	GCA_002749615	4.58	69.3
Caulobacter zeae	410	PJRS01	GCA_002858925	5.39	69.0
Caulobacter radicis	695	QDKO01	GCA_003094615	5.57	69.1
Caulobacter henricii	CB4	-	GCA_001414055	3.96	65.8
Caulobacter endophyticus	774	QDKQ01	GCA_003116815	5.42	68.7
Caulobacter hibisci	KACC 18849	JADWOX01	GCA_016135805	5.57	68.8
Caulobacter soli	Ji-3-8	-	GCA_011045195	5.68	68.1
Phenylobacterium zucineum	HLK1	-	GCA_000017265	4.38	71.0
Phenylobacterium immobile	ATCC 35973	CVJQ01	GCA_001375595	3.33	66.7
Phenylobacterium parvum	HYN0004	-	GCA_003150835	2.83	69.9

Phenylobacterium soli	LX32	QFYQ01	GCA_003254475	4.02	70.1
Phenylobacterium haematophilum	DSM 21793	JACIDK01	GCA_014196295	4.43	67.9
Phenylobacterium deserti	YIM 73061	QFYR01	GCA_003254705	3.88	68.2
Phenylobacterium kunshanense	BUT-10	QFYS01	GCA_003254525	4.18	69.2
Phenylobacterium hankyongense	HKS-05	QFYP01	GCA_003254505	3.84	70.3
Terricaulis silvestris	0127_4	-	GCA_009792355	3.86	63.5

Table S4: The distribution of antimicrobial resistance genes of *Brevundimonas* spp.

**Species**	**Genes**	**Detection Criteria**	**AMR**	**Drug**	**Resistance**	**Identity of Matching Region (%)**
Brevundimonas_abyssalis_TAR_001	NA	protein homolog model	NA	NA	NA	NA
Brevundimonas_alba_DSM_4736	*adeF*	protein homolog model	resistance-nodulation-cell division (RND) antibiotic efflux pump	fluoroquinolone antibiotic, tetracycline antibiotic	antibiotic efflux	50.43
Brevundimonas_aurantiaca_DSM_4731.fasta	*adeF*	protein homolog model	resistance-nodulation-cell division (RND) antibiotic efflux pump	fluoroquinolone antibiotic, tetracycline antibiotic	antibiotic efflux	52.05
Brevundimonas_aveniformis_DSM_17977	*adeF*	protein homolog model	resistance-nodulation-cell division (RND) antibiotic efflux pump	fluoroquinolone antibiotic, tetracycline antibiotic	antibiotic efflux	50
Brevundimonas_bacteroides_DSM_4726	*adeF*	protein homolog model	resistance-nodulation-cell division (RND) antibiotic efflux pump	fluoroquinolone antibiotic, tetracycline antibiotic	antibiotic efflux	49.25
Brevundimonas_basaltis_DSM_25335	*adeF*	protein homolog model	resistance-nodulation-cell division (RND) antibiotic efflux pump	fluoroquinolone antibiotic, tetracycline antibiotic	antibiotic efflux	51.08
Brevundimonas_bullata_HAMBI_262	*adeF*	protein homolog model	resistance-nodulation-cell division (RND) antibiotic efflux pump	fluoroquinolone antibiotic, tetracycline antibiotic	antibiotic efflux	50.19

Brevundimonas_denitrificans_TAR_002	NA	protein homolog model	NA	NA	NA	NA
Brevundimonas_diminuta_ATCC_11568	*tet(C)*	protein homolog model	major facilitator superfamily (MFS) antibiotic efflux pump	tetracycline antibiotic	antibiotic efflux	100
	adeF	protein homolog model	resistance-nodulation-cell division (RND) antibiotic efflux pump	fluoroquinolone antibiotic, tetracycline antibiotic	antibiotic efflux	49.35
Brevundimonas_fluminis_LA_55	NA	protein homolog model	NA	NA	NA	NA
Brevundimonas_halotolerans_MCS24	*adeF*	protein homolog model	resistance-nodulation-cell division (RND) antibiotic efflux pump	fluoroquinolone antibiotic, tetracycline antibiotic	antibiotic efflux	50.67
Brevundimonas_intermedia_B_10	*adeF*	protein homolog model	resistance-nodulation-cell division (RND) antibiotic efflux pump	fluoroquinolone antibiotic, tetracycline antibiotic	antibiotic efflux	51.9
Brevundimonas_lenta_DSM_23960	*adeF*	protein homolog model	resistance-nodulation-cell division (RND) antibiotic efflux pump	fluoroquinolone antibiotic, tetracycline antibiotic	antibiotic efflux	50.24
Brevundimonas_lutea_NS26	*adeF*	protein homolog model	resistance-nodulation-cell division (RND) antibiotic efflux pump	fluoroquinolone antibiotic, tetracycline antibiotic	antibiotic efflux	50.57
Brevundimonas_mediterranea_DSM_14878	*adeF*	protein homolog model	resistance-nodulation-cell division (RND) antibiotic efflux pump	fluoroquinolone antibiotic, tetracycline antibiotic	antibiotic efflux	50.86
Brevundimonas_naejangsanensis_DSM_23858	*sul2*	protein homolog model	sulfonamide resistant sul	sulfonamide antibiotic	antibiotic target replacement	100
	tet(D)	protein homolog model	major facilitator superfamily (MFS) antibiotic efflux pump	tetracycline antibiotic	antibiotic efflux	51.9
	tet(G)	protein homolog model	major facilitator superfamily (MFS) antibiotic efflux pump	tetracycline antibiotic	antibiotic efflux	94.13
	adeF	protein homolog model	resistance-nodulation-cell division (RND) antibiotic efflux pump	fluoroquinolone antibiotic, tetracycline antibiotic	antibiotic efflux	49.16

Brevundimonas_nasdae_JCM_11415	*adeF*	protein homolog model	resistance-nodulation-cell division (RND) antibiotic efflux pump	fluoroquinolone antibiotic, tetracycline antibiotic	antibiotic efflux	51.81
Brevundimonas_sp_novCHPC1_3453	*adeF*	protein homolog model	resistance-nodulation-cell division (RND) antibiotic efflux pump	fluoroquinolone antibiotic, tetracycline antibiotic	antibiotic efflux	48.74
Brevundimonas_subvibrioides_ATCC_15264	*adeF*	protein homolog model	resistance-nodulation-cell division (RND) antibiotic efflux pump	fluoroquinolone antibiotic, tetracycline antibiotic	antibiotic efflux	49.35
Brevundimonas_terrae_DSM_17329	*adeF*	protein homolog model	resistance-nodulation-cell division (RND) antibiotic efflux pump	fluoroquinolone antibiotic, tetracycline antibiotic	antibiotic efflux	48.84
Brevundimonas_vancanneytii_NCTC_9239	*adeF*	protein homolog model	resistance-nodulation-cell division (RND) antibiotic efflux pump	fluoroquinolone antibiotic, tetracycline antibiotic	antibiotic efflux	49.35
Brevundimonas_variabilis_DSM_4737	*adeF*	protein homolog model	resistance-nodulation-cell division (RND) antibiotic efflux pump	fluoroquinolone antibiotic, tetracycline antibiotic	antibiotic efflux	50.67
Brevundimonas_vesicularis_NBRC_12165	*adeF*	protein homolog model	resistance-nodulation-cell division (RND) antibiotic efflux pump	fluoroquinolone antibiotic, tetracycline antibiotic	antibiotic efflux	51.62
Brevundimonas_viscosa_CGMCC_1_10683	*adeF*	protein homolog model	resistance-nodulation-cell division (RND) antibiotic efflux pump	fluoroquinolone antibiotic, tetracycline antibiotic	antibiotic efflux	51.33

Table S5: Detailed information of genomic islands at the cross-species level

**Strain**	**GI**	**Length (bp)**	**Cover (%)**	**Identity (%)**	**Match species**	**Accession No.**
B. aurantiaca DSM 4731	229395..238057	8662	100	100	*Brevundimonas* sp. DS20	CP012897.1
	262111..266323	4212	100	100	*Brevundimonas* sp. GW460-12-10-14-LB2	CP015511.1
	271357..275913	4556	100	99.96	*Brevundimonas* sp. GW460-12-10-14-LB2	CP015511.1
	281719..286260	4541	100	100	*Brevundimonas* sp. GW460-12-10-14-LB2	CP015511.1
	778299..801433	23134	86	97.28	*Brevundimonas* sp. Bb-A	CP045456.1

	1017060..1034220	17160	90	98.23	*Brevundimonas* sp. Bb-A	CP045456.1
	1442167..1452887	10720	100	98.83	*Brevundimonas* sp. Bb-A	CP045456.1
	1442631..1456306	13675	100	98.16	*Brevundimonas* sp. Bb-A	CP045456.1
	1505610..1512848	7238	100	99.18	*Brevundimonas* sp. Bb-A	CP045456.1
	1888999..1894648	5649	100	99.98	*Brevundimonas* sp. DS20	CP012897.1
	1906304..1968254	61950	100	99.97	*Brevundimonas* sp. DS20	CP012897.1
	1960974..1969343	8369	100	100	*Brevundimonas* sp. DS20	CP012897.1
B. bullata HAMBI_262	1498930..1512170	13240	100	97.97	*Brevundimonas naejangsanensis* FS1091	CP038027.1
	1593972..1600224	6252	100	99.36	*Brevundimonas* sp. scallop	CP039382.1
B. diminuta ATCC 11568	429195..435759	6564	100	94.88	*Brevundimonas naejangsanensis* FS1091	CP038027.1
	2030727..2040378	9651	94	98.34	*Brevundimonas vancanneytii* NCTC9239	LR588407.1
	2534852..2542168	7316	100	98.58	*Brevundimonas* sp. LVF1	CP062006.1
B. intermedia B-10	65421..69792	4371	100	99.95	*Brevundimonas vesicularis* FDAARGOS_289	CP022048.2
	3335481..3340816	5335	100	99.34	*Brevundimonas mediterranea* D151-2-6	CP048751.1
B. lenta DSM 23960	2713044..2718536	5492	100	99.85	*Brevundimonas* sp. LVF2	CP062222.1
B. mediterranea DSM 14878	3176907..3181278	4371	100	100	*Brevundimonas vesicularis* FDAARGOS_289	CP022048.2
B. nasdae JCM 11415	2867279..2873454	6175	100	100	*Brevundimonas naejangsanensis* B1	CP015614.1
Brevundimonas sp. nov. CHPC 1.3453	76674..106911	30237	100	100	*Brevundimonas subvibrioides* ATCC 15264	CP002102.1
	223016..235718	12702	100	100	*Brevundimonas subvibrioides* ATCC 15264	CP002102.1
	1148588..1154042	5454	100	100	*Brevundimonas subvibrioides* ATCC 15264	CP002102.1
	1154902..1166364	11462	100	100	*Brevundimonas subvibrioides* ATCC 15264	CP002102.1
	1224734..1257413	32679	100	100	*Brevundimonas subvibrioides* ATCC 15264	CP002102.1
	1359246..1375567	16321	100	100	*Brevundimonas subvibrioides* ATCC 15264	CP002102.1
	1387459..1395892	8433	100	100	*Brevundimonas subvibrioides* ATCC 15264	CP002102.1
	1609864..1627383	17519	100	100	*Brevundimonas subvibrioides* ATCC 15264	CP002102.1
	1615975..1621030	5055	100	100	*Brevundimonas subvibrioides* ATCC 15264	CP002102.1

	1973945..1996939	22994	100	100	*Brevundimonas subvibrioides* ATCC 15264	CP002102.1
	2011416..2028195	16779	100	100	*Brevundimonas subvibrioides* ATCC 15264	CP002102.1
	2043541..2047710	4169	100	100	*Brevundimonas subvibrioides* ATCC 15264	CP002102.1
	2638755..2648483	9728	100	100	*Brevundimonas subvibrioides* ATCC 15264	CP002102.1
B. vancanneytii NCTC 9239	724237..729871	5634	100	97.11	*Brevundimonas diminuta* BZC3	LR588407.1
	1033149..1039491	6342	100	98.57	*Brevundimonas diminuta* FDAARGOS_1026	CP066026.1
	1306437..1315931	9494	100	98.8	*Brevundimonas diminuta* BZC3	CP021995.1
	1307777..1315188	7411	100	98.89	*Brevundimonas diminuta* BZC3	CP021995.1
	1316354..1328644	12290	100	99.17	*Brevundimonas diminuta* BZC3	CP021995.1
	1301945..1306325	4380	100	97.9	*Brevundimonas diminuta* BZC3	CP021995.1
	121930..127035	5105	93	98.97	*Brevundimonas diminuta* BZC3	CP021995.1
	628830..633043	4213	99	97.53	*Brevundimonas diminuta* BZC3	CP021995.1
	720318..725202	4884	100	95.2	*Brevundimonas diminuta* BZC3	CP021995.1
	623977..628717	4740	100	97.26	*Brevundimonas diminuta* ATCC(B) 19146	CP035093.1
	1324879..1330372	5493	100	99.11	*Brevundimonas diminuta* BZC3	CP021995.1
	1334266..1341816	7550	100	98.82	*Brevundimonas diminuta* BZC3	CP021995.1
	1340612..1346388	5776	100	98.77	*Brevundimonas diminuta* BZC3	CP021995.1
	1434325..1438815	4490	95	98.04	*Brevundimonas diminuta* ATCC(B) 19146	CP035093.1
	1458253..1469846	11593	97	99.95	*Brevundimonas diminuta* ATCC(B) 19146	CP035093.1
	1475552..1480919	5367	100	100	*Brevundimonas diminuta* ATCC(B) 19146	CP035093.1
	1485607..1490091	4484	100	99.96	*Brevundimonas diminuta* ATCC(B) 19146	CP035093.1
	1492510..1497232	4722	100	99.98	*Brevundimonas diminuta* ATCC(B) 19146	CP035093.1
	1533713..1542139	8426	100	99.98	*Brevundimonas diminuta* ATCC(B) 19146	CP035093.1
	1539521..1545656	6135	100	99.97	*Brevundimonas diminuta* ATCC(B) 19146	CP035093.1
	1652057..1660324	8267	100	99.08	*Brevundimonas diminuta* BZC3	CP021995.1
	1718237..1723127	4890	99	99.43	*Brevundimonas diminuta* ATCC(B) 19146	CP035093.1

	1718406..1723709	5303	100	99.38	*Brevundimonas diminuta* ATCC(B) 19146	CP035093.1
	1900190..1905040	4850	100	97.88	*Brevundimonas diminuta* BZC3	CP021995.1
	1905107..1925371	20264	100	97.57	*Brevundimonas diminuta* ATCC(B) 19146	CP035093.1
	2186576..2201615	15039	100	97.57	*Brevundimonas diminuta* BZC3	CP021995.1
	2201982..2215161	13179	99	97.47	*Brevundimonas diminuta* BZC3	CP021995.1
	2228932..2235425	6493	100	98	*Brevundimonas diminuta* BZC3	CP021995.1
	2231296..2236012	4716	100	98.28	*Brevundimonas diminuta* BZC3	CP021995.1
	2231863..2236213	4350	100	98.4	*Brevundimonas diminuta* BZC3	CP021995.1
	2237963..2242203	4240	91	99.9	*Brevundimonas naejangsanensis* FS1091	CP038027.1
	2245183..2253220	8037	100	99.83	*Brevundimonas naejangsanensis* FS1091	CP038027.1
	2252129..2284477	32348	98	99.86	*Brevundimonas naejangsanensis* FS1091	CP038027.1
	2260927..2271990	11063	100	99.93	*Brevundimonas naejangsanensis* FS1091	CP038027.1
	2279619..2284477	4858	90	99.76	*Brevundimonas naejangsanensis* FS1091	CP038027.1
	2518242..2523211	4969	100	97.95	*Brevundimonas diminuta* ATCC(B) 19146	CP035093.1
	2601499..2606378	4879	100	97.79	*Brevundimonas diminuta* ATCC(B) 19146	CP035093.1
	2930438..2936270	5832	99	93.55	*Brevundimonas diminuta* BZC3	CP021995.1
B. vesicularis NBRC 12165	661536..713462	51926	92	98.34	*Brevundimonas* sp. DS20	CP012897.1
	2299291..2304112	4821	100	99.2	*Brevundimonas* sp. DS20	CP012897.1
	2311480..2331649	20169	100	99.93	*Brevundimonas* sp. DS20	CP012897.1
	2347678..2357542	9864	100	99.93	*Brevundimonas* sp. DS20	CP012897.1
	2380398..2385646	5248	99	96.61	*Brevundimonas* sp. SGAir0440	CP039435.1
	3123744..3137491	13747	92	97.14	*Brevundimonas* sp. GW460-12-10-14-LB2	CP015511.1

Table S6: Detailed information of genomic islands at the cross-genus level

Strain	GI	Total Length (bp)	Coverage (%)	Identity (%)	Cover length	Match species	Accession No.
B. abyssalis TAR-001	1197565..1239942	42377	86.2	98.9	36529	*Defluviimonas alba* cai42	CP012661.1
B. alba DSM 4736	977926..1006081	28155	19.4	84.1	5462	*Phenylobacterium zucineum* HLK1	CP000747.1
B. aveniformis DSM 17977	178193..212713	34520	14.8	89.0	5109	*Rhizobium* sp. CIAT894	CP020947.1
B. bullata HAMBI_262	1220059..1226273	6214	100	87.4	6214	*Parastrongyloides trichosuri* KNP	LM523251.1
	1471904..1488157	16253	50.5	83.1	8208	*Ochrobactrum* sp. PW1	LC171366.1
	1357780..1367972	10192	52.2	99.2	5320	*Phenylobacterium zucineum* HLK1	CP000748.1
	3424605..3484004	59399	35.5	81.3	21087	*Sphingopyxis terrae* YC-JH3	CP050249.1
B. denitrificans TAR-002	858935..895243	36308	89.7	98.8	32568	*Defluviimonas alba* cai42	CP012661.1
B. fluminis LA55	2633181..2639789	6608	76.8	97.7	5075	*Cupriavidus metallidurans* FDAARGOS_675	CP046331.1
B. mediterranea DSM 14878	927569..989509	61940	29.2	96.0	18086	*Caulobacter* sp. K31	CP000929.1
B. naejangsanensis DSM 23858	2460714..2505385	44671	15.3	97.4	6835	*Azorhizobium caulinodans* ORS 571	AP009384.1
	2482588..2527974	45386	63.4	98.7	28775	*Brucella anthropi* PBO	CP064063.1
	2519330..2529171	9841	100	100.0	9841	*Brucella anthropi* PBO	CP064063.1
B. nasdae JCM 11415	1899443..1922387	22944	25.3	98.8	5805	*Parvibaculum lavamentivorans* DS-1	CP000774.1
	1907933..1916263	8330	66.2	98.8	5514	*Parvibaculum lavamentivorans* DS-1	CP000774.1
	3608231..3700829	92598	27.2	97.5	25187	*Parvibaculum lavamentivorans* DS-1	CP000774.1
B. vancanneytii NCTC 9239	3110252..3151154	40902	19.0	89.3	7771	*Rhodobacter* sp. LPB0142	CP017781.1

Table S7: Details of phage/prophage prediction of *Brevundimonas* species

No.	Species	Strain	Completeness	Most Common Phage	Region Length (Kb)	Score	Total Proteins	GC %	Accession No.
1	*Brevundimonas alba*	DSM 4736^T	questionable	PHAGE_Paraco_vB_PmaS_IMEP1	17.9	70	22	68.18	NC_026608
2	*Brevundimonas aveniformis*	DSM 17977^T	questionable	PHAGE_Paraco_vB_PmaS_IMEP1	16.0	70	21	67.29	NC_026608
3	*Brevundimonas bacteroides*	DSM 4726^T	questionable	PHAGE_Paraco_vB_PmaS_IMEP1	20.8	70	26	70.34	NC_026608
4	*Brevundimonas diminuta*	ATCC 11568^T	questionable	PHAGE_Acinet_Bphi_B1251	11.6	70	17	63.97	NC_019541
			questionable	PHAGE_Pseudo_Dobby	38.2	70	57	64.92	NC_048109
5	*Brevundimonas fluminis*	LA-55^T	questionable	PHAGE_Paraco_vB_PmaS_IMEP1	17.4	80	22	72.26	NC_026608
6	*Brevundimonas halotolerans*	MCS24^T	questionable	PHAGE_Salmon_118970_sal3	16.1	70	20	69.62	NC_031940
7	*Brevundimonas nasdae*	JCM 11415^T	questionable	PHAGE_Paraco_vB_PmaS_IMEP1	26.1	80	31	69.06	NC_026608
			questionable	PHAGE_Sinorh_phiLM21	14.0	80	20	65.04	NC_029046
8	*Brevundimonas* sp. nov.	CHPC 1.3453^T	intact	PHAGE_Ralsto_RsoM1USA	24.2	150	31	61.42	NC_049432
9	*Brevundimonas vancanneytii*	NCTC 9239	intact	PHAGE_Stx2_c_Stx2a_F451	14.4	120	15	62.84	NC_049924
			questionable	PHAGE_Escher_RCS47	12.4	70	12	62.50	NC_042128
			questionable	PHAGE_Staphy_SPbeta_like	15.8	80	14	64.17	NC_029119
10	*Brevundimonas vesicularis*	NBRC 12165^T	questionable	PHAGE_Paraco_vB_PmaS_IMEP1	22.1	70	25	69.49	NC_026608
11	*Brevundimonas viscosa*	CGMCC 1.10683^T	intact	PHAGE_Sinorh_phiLM21	48.5	150	61	68.46	NC_029046
